# Histamine H_3_ Receptor-Mediated Signaling Protects Mice from Cerebral Malaria

**DOI:** 10.1371/journal.pone.0006004

**Published:** 2009-06-23

**Authors:** Walid Beghdadi, Adeline Porcherie, Bradley S. Schneider, Séverine Morisset, David Dubayle, Roger Peronet, Michel Dy, Jacques Louis, Jean-Michel Arrang, Salaheddine Mécheri

**Affiliations:** 1 Unité des Réponses Précoces aux Parasites et Immunopathologie, Institut Pasteur, Paris, France; 2 INSERM Unité de Neurobiologie et Pharmacologie Moléculaire, Centre de Psychiatrie et Neurosciences, Paris, France; 3 Université Paris Descartes - CNRS UMR 8119, Paris, France; 4 Cytokines, Hématopoïèse et Réponse Immune, CNRS UMR 8147 Hôpital Necker, Paris, France; Federal University of São Paulo, Brazil

## Abstract

**Background:**

Histamine is a biogenic amine that has been shown to contribute to several pathological conditions, such as allergic conditions, experimental encephalomyelitis, and malaria. In humans, as well as in murine models of malaria, increased plasma levels of histamine are associated with severity of infection. We reported recently that histamine plays a critical role in the pathogenesis of experimental cerebral malaria (CM) in mice infected with *Plasmodium berghei* ANKA. Histamine exerts its biological effects through four different receptors designated H1R, H2R, H3R, and H4R.

**Principal Findings:**

In the present work, we explored the role of histamine signaling via the histamine H3 receptor (H3R) in the pathogenesis of murine CM. We observed that the lack of H3R expression (H3R^−/−^ mice) accelerates the onset of CM and this was correlated with enhanced brain pathology and earlier and more pronounced loss of blood brain barrier integrity than in wild type mice. Additionally *tele*-methylhistamine, the major histamine metabolite in the brain, that was initially present at a higher level in the brain of H3R^−/−^ mice was depleted more quickly post-infection in H3R^−/−^ mice as compared to wild-type counterparts.

**Conclusions:**

Our data suggest that histamine regulation through the H3R in the brain suppresses the development of CM. Thus modulating histamine signaling in the central nervous system, in combination with standard therapies, may represent a novel strategy to reduce the risk of progression to cerebral malaria.

## Introduction

The main cellular reserves of histamine in peripheral tissues are mast cells and basophils. Histamine release is involved in the pathogenesis of various inflammatory reactions [1], [2]. In the central nervous system (CNS), histamine also acts as a neurotransmitter that is released by histaminergic neurons. The cell bodies of histaminergic neurons are located exclusively in the tuberomammillary nucleus of the posterior hypothalamus and project their axons in a highly divergent manner to many cerebral areas including the hypothalamus, thalamus, cerebral cortex, amygdala, and septum [3], [4], [5], [6]. Four histamine receptors have been identified and termed H1-, H2-, H3-, and H4 receptors [3], [7] all of which are G-protein coupled receptors. The H1 receptor (H1R) mediates most of the proinflammatory effects of histamine. Anti-inflammatory and immunosuppressive effects of histamine, such as inhibition of polymorphonuclear chemotaxis [8], and interleukin (IL)-12 secretion by monocytes, and induction of IL-10 production [9], are mainly dependent on stimulation of the H2 receptor (H2R), which is positively coupled to the adenylyl cyclase pathway. Unlike the other histamine receptors, H4 receptor (H4R) is predominantly expressed on hematopoietic cells [10], [11], [12], and H4R agonists were shown to induce chemotaxis of mast cells and eosinophils [13] as well as the production of IL-16 by T cells [14]. Recently, using a murine model of allergic asthma, it was demonstrated that H4R stimulation induces inhibition of airway resistance and inflammation via a CD25^+^FoxP3^+^ T regulatory cell-dependent mechanism [15].

In contrast to H1R, H2R, and H4R, H3R are mainly expressed in neurons of the central and peripheral nervous system [16]. Presynaptic H3Rs located on histaminergic nerve endings, act as autoreceptors to control the synthesis and release of histamine [5], [17]. Presynaptic H3Rs also act as heteroreceptors that influence the release of other neurotransmitters including dopamine, γ-aminobutyric acid, noradrenaline, acetylcholine, serotonin and tachykinins [18]. There is also some evidence for the existence of H3R in the gastrointestinal tract where it exerts negative control on gastric acid secretion [19]. Given the location of its expression, it has been suggested that H3R, by modulating the brain histaminergic tone, mediate various CNS functions affecting a variety of behaviors. In this context, histamine was shown to play a critical role in the regulation of the arousal state [20], locomotor activity [21], food intake [22], memory and cognition [23].

With regard to neurological disorders, mice lacking H3R (H3R^−/−^) develop more severe experimental allergic encephalomyelitis (EAE) with a marked increase of blood brain barrier permeability and an increased expression of macrophage-inflammatory protein (MIP)-2 and interferon-inducible protein-10 (IP-10/CXCL10) on peripheral T cells. Furthermore, increased tissue levels of histamine correlate with the onset of EAE [24], [25], [26]. Experimental cerebral malaria (CM) in mice is a severe pathological condition resulting from infection with particular strains of *Plasmodium* parasites, namely *Plasmodium berghei* ANKA strain (*Pb* ANKA). Increased levels of histamine in plasma and tissue is associated with the severity of human infection with *P. falciparum* and in animal models of malaria [27]. In a recent study, using pharmacological and genetic approaches, we demonstrated that histamine plays a critical role in malaria pathogenesis in mice [28]. We found that histamine signaling through H1R and H2R increases the susceptibility of mice to infection with lethal strains of *P. berghei* [28]. Furthermore, mice genetically deficient for the histidine decarboxylase (HDC^−/−^) gene, and thus lacking histamine, were highly resistant to severe malaria whether infected by mosquito bites or via injection of infected erythrocytes. To investigate the role of H3R signaling in the regulation of the inflammatory response in the brain during malaria, we studied *Pb* ANKA-induced CM in H3R^−/−^ mice. Herein, we report an accelerated onset of cerebral malaria and increased blood brain barrier (BBB) permeability in H3R^−/−^ mice as compared to wild-type mice.

## Results

### Lack of H3R expression accelerates the onset of CM

**Figure 1:**
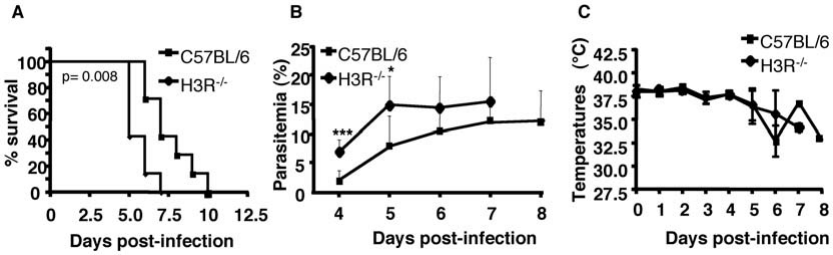
Role of H3R in *Plasmodium berghei* infection. H3R^−/−^ and C57BL/6 mice were inoculated with 10^6^ infected erthrocytes with *Pb* ANKA. Kaplan-Meier Survival plots (A), parasitemia (B), and body temperature (C) were recorded. Significant differences in mortality/survival were observed between C57BL/6 and H3R^−/−^ mice using the log-rank test (n = 6, p = 0.0092). Differences in parasitemia between groups are significant at day 4 and 5 (Mann-whitney test, *** p = 0.0015, and *p = 0.0125, respectively), values represent mean±s.d. Data shown are representative of four independent experiments.

Wild type C57BL/6 mice display neurological signs characteristic of CM within 6–11 days after infection with parasites from the *Pb* ANKA strain, and death usually occurs within 24 h after the onset of these signs [29]. Those mice that do not succumb during this period will die later due to hyperparasitemia and anemia. Based on previous data showing that the lack of histamine production confers resistance to CM and given that H3R signaling inhibits the synthesis and the release of histamine by histaminergic neurons [5], [17], we hypothesized that the uncontrolled histamine release by histaminergic neurons, resulting from a deficiency in H3R signaling, would be detrimental to the host during malaria disease. To experimentally assess this hypothesis we studied the role of the H3R in malaria pathogenesis, by monitoring the parasitemia and death over time in H3R^−/−^ mice inoculated with 10^6^ infected erythrocytes. As shown in figure 1A, death occurred significantly earlier (n = 6, p = 0.0092) in H3R^−/−^ mice than in similarly infected C57BL/6 control mice (median survival: day 6 and 9, respectively). A significantly higher parasitemia was observed in H3R^−/−^ mice at day 4 (p = 0.0015) and day 5 (p = 0.0124) post-infection (Fig 1B). Decreasing the infectious dose of RBC to 10^5^ per mouse did not alter the phenotypic difference between the two mouse strains (data not shown), suggesting that parasitemia does not represent the critical parameter for accelerated disease expression and mortality in H3R^−/−^ mice. Among the critical signs observed during CM is the drop in body temperature. In this regard, both H3R^−/−^ and C57BL/6 displayed a decline in body temperature starting from day 5 and no significant difference was observed between groups (Fig 1C).

### Accelerated loss of BBB integrity in infected H3R^−/−^ mice

**Figure 2:**
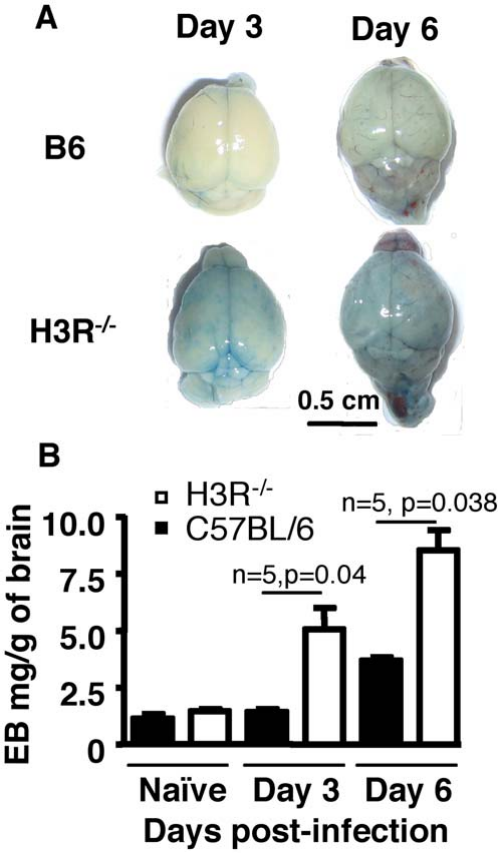
Accelerated loss of blood brain barrier in the brain of H3R^−/−^ mice. (A), Wild-type C57BL/6 mice and H3R^−/−^ mice (3 mice per group) were inoculated intraperitoneally with 10^6^ infected erythrocytes per mouse of *Pb* ANKA strain. At day 3 and day 6 post-infection, mice were injected with a solution of Evans blue dye and 1 h later were perfused with PBS and macroscopic observation of the brains was made. Brains from uninfected mice were used as controls. (B) Measurement of Evans blue dye extravasation by spectrophotometry. Data are representative of two experiments. Significant differences were determined by Mann-Whitney test.

The rapid onset of CM in H3R^−/−^ mice prompted us to determine whether or not the H3R signaling affects BBB permeability during infection with *Pb* ANKA. We compared the BBB permeability of C57BL/6 and H3R^−/−^ mice at various time points after inoculation with 10^6^ infected erythrocytes. As shown in figure 2B, the loss of BBB integrity occurred earlier at day 3 and at a higher magnitude in H3R^−/−^ mice than in C57BL/6 mice (n = 5, p = 0.04) with almost no visible Evans blue dye extravasation in the brains from C57BL/6 mice (Fig. 2A). At day 6, the loss of BBB integrity became more visible both in C57BL/6 and H3R^−/−^ mice (Fig. 2A) with a greater BBB permeability index in H3R^−/−^ mice (n = 5, p = 0.038) (Fig. 2B). The earlier loss of BBB integrity in infected H3R^−/−^ mice is consistent with the accelerated onset of mortality (Fig. 1A).

**Figure 3:**
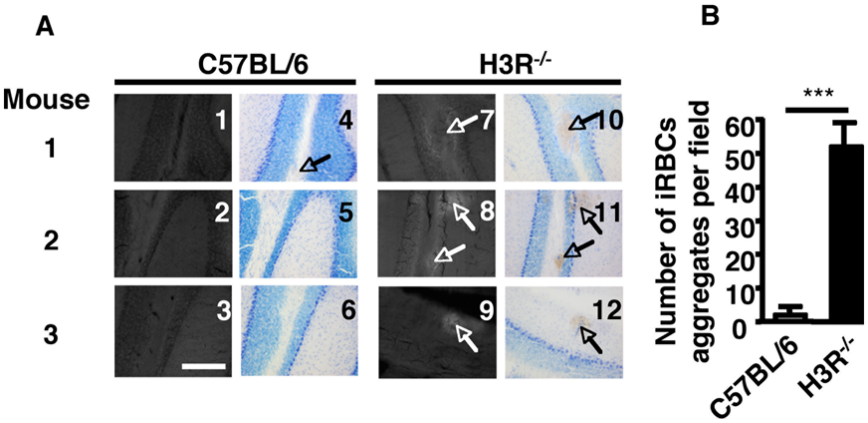
Formation of hemorrhagic lesions occurs earlier in the brain of H3R^−/−^ mice. Wild-type C57BL/6 mice and H3R^−/−^ mice (3 mice per group) were inoculated intraperitoneally with 10^6^ *Pb* ANKA-infected erythrocytes per mouse. At day 5 post-infection, mice were perfused with PBS then PFA 4% and brain sections were made. (A) Brains Sections stained with May-Grünwald Giemsa. Blood-stage parasites associated with sequestered erythrocytes (panels 10, 11, and 12) in the brain could be visualized by fluorescence (panels, 7, 8, and 9) because of their expression of GFP only in H3R^−/−^ mice (white shadow indicated by arrows). At this time, no deposition of fluorescent parasites could be observed in brain sections from uninfected (not shown) and only scarce number of aggregates could be detected in infected C57BL/6 mice (arrow in panel 4 for mouse 1). Data are representative of three mice per group. (B), The density of erythrocyte aggregates in brain sections of infected H3R^−/−^ and C57BL/6 mice were expressed as the average of aggregates in three sections and counted in 250 consecutive microscopic fields per section at magnification 250×. These data are from two different experiments. *** Significant difference was obtained between infected H3R^−/−^ and C57BL/6 mice using Mann-Whitney test (p = 0.0003). Bar, 100 µm.

The neurological signs that characterize CM are generally accompanied by the sequestration of infected erythrocytes in the cerebral vasculature [30], [31]. Histological analysis of brain sections obtained from naïve or at day 5 post-infection of C57BL/6 did show very discrete erythrocyte aggregates observed in sections from olfactory bulbs and other anatomical parts of the brain (mouse 1, panel 4, Fig 3A). Deposition of erythrocyte aggregates, representing either erythrocyte sequestration or cerebral hemorrhages, becomes more significant in C57BL/6 mice only by day 7 post-infection (data not shown, and [28]. In sharp contrast, higher amounts of aggregates of much larger size were found in identically infected H3R^−/−^ mice (Fig 3A). Importantly, these aggregates consisted of infected erythrocytes as shown by fluorescence from GFP-expressing parasites used for infection (Fig 3A). Quantification of brain lesions, expressed as the number of erythrocyte aggregates in 100 consecutive microscopic fields from each of the 3 histological sections made from the cerebellum, confirmed a significantly larger number of infected erythrocyte aggregates in H3R^−/−^ mice as compared to C57BL/6 mice (Fig. 3B). These data show that the loss of BBB integrity in infected H3R^−/−^ mice is associated with an accelerated formation of hemorrhagic lesions and with earlier development of CM.

### CM expression is associated with infiltrates of inflammatory cells in the brain

**Figure 4:**
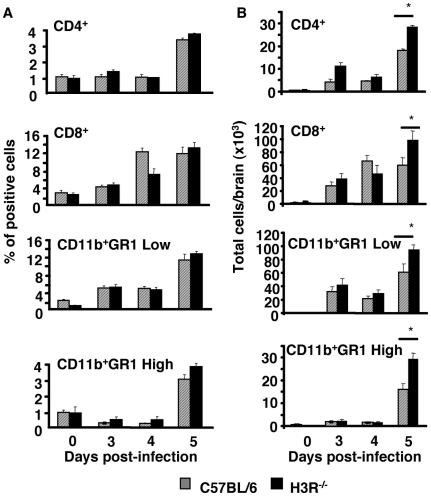
Characterization of brain infiltrating cells during infection with *Pb* ANKA parasites. At indicated time points post-infection, brains from C57BL/6 and H3R^−/−^ mice infected with 10^6^ infected erythrocytes per mouse of *Pb* ANKA strain were taken and leukocytes associated with cerebral tissue were analyzed for the presence of CD4^+^ and CD8^+^ T cells and other inflammatory cells. Data are expressed as a percentage of total cell population (A) and absolute cell numbers per brain (B). Six mice per group were used. Values represent the mean±standard deviation of two experiments. (B) Differences between C57BL/6 and H3R^−/−^ mice are significant at day 5 for total CD4^+^ and CD8^+^ T cells (Mann-Whitney test, *p = 0.04). Differences in total neutrophils/inflammatory monocytes (CD11b^+^ GR1 High cells) and (CD11b^+^ GR1 Low cells) between C57BL/6 and H3R^−/−^ mice are significant at day 5 (Mann-Whitney test, *p = 0.04, and p = 0.046, respectively).

To determine the number of adherent cells present in the brain, nonadherent cells were removed by extensive perfusion of the brain of anesthetized mice before sacrifice at various times after infection with *Pb* ANKA. The perfused brains were removed and the cells dissociated. Then the adherent leukocytes were isolated, immunolabeled, and quantified by FACS. Given that recruitment of CD4^+^ and CD8^+^ T cells in the brain is a hallmark of cerebral malaria [28], [29], a comparative analysis of CD4^+^ and CD8^+^ T cells infiltrating the brain was performed in H3R^−/−^ and C57BL/6 mice. The data were expressed as percentage of sequestered cells (Fig. 4A) and as the absolute number of adherent cells (Fig. 4B). We could observe a progressive sequestration of both CD4^+^ and CD8^+^ T cells starting 3 days post-infection in both mouse strains. At day 5, however, a higher sequestration of CD4^+^ and CD8^+^ T cells was observed in H3R^−/−^ mice as compared to C57BL/6 mice (p = 0.04). FACS phenotyping, extended to other inflammatory cell lineages, revealed an increase in the numbers of macrophages/monocytes (CD11b^+^-GR1 low) with a significantly higher sequestration in H3R^−/−^ mice that occurred relatively late at day 5 post-infection (p = 0.046). Analysis of CD11b^+^-GR1 high cells, likely representing neutrophils or inflammatory monocytes as reported previously [32], also indicates a progressive increase starting from day 3 post-infection with significantly higher levels in H3R^−/−^ mice as compared to C57BL/6 mice (p = 0.04). More detailed analysis of this cell population is described in supporting figure 1.

### *tele*-Methylhistamie contents in the brains of *Pb* ANKA-infected mice

**Figure 5:**
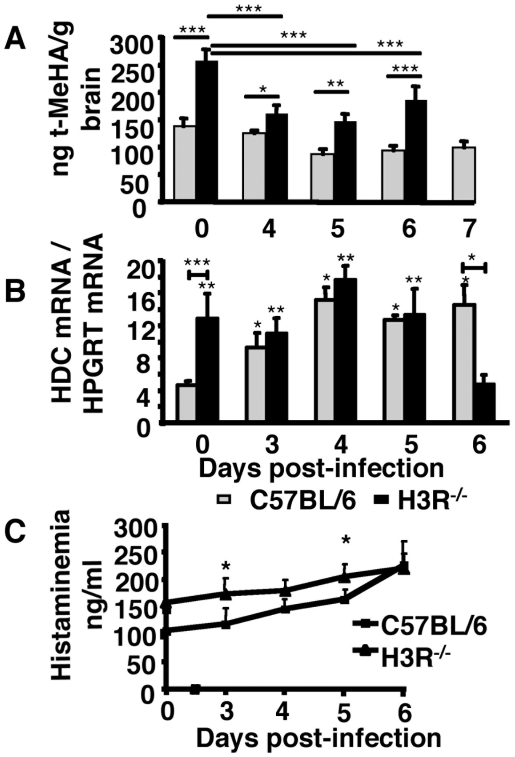
Regulation of histamine metabolism upon infection with *Pb* ANKA. (A) C57BL/6 and H3R^−/−^ mice were sacrificed at indicated time points after receiving blood stage parasites of *Pb* ANKA inoculated intravenously, and brain extracts were prepared for t-MeHA measurements. Differences in t-MeHA level between groups are significant (Mann-Whitney test, n = 5, *** p<0.001, ** 0.001<p<0.01, and * 0.01<p<0.05). In C57Bl/6 mice, significant differences in t-MeHA level appeared day 5 post-inoculation (Kruskal Wallis test, p = 0.0061 followed by Dunn's Multiple Comparaison test, *** p<0.0001). In H3R KO mice, significant differences in t-MeHA level appeared day 4 post inoculation (Kruskal Wallis test, p = 0.008 followed by Dunn's Multiple Comparaison test, *** p = 0.0002). The t-MeHA level for H3R^−/−^ mice on day 7 is absent due to the high level of mortality by that time point. (B) Transcription of the HDC gene in the brain (n = 6/group) at different time points post-infection as evaluated by real-time RT-PCR. mRNA expression was normalized relative to hypoxanthinephophoribosyltransferase expression for each mouse strain. * indicates that differences are significant (Mann-Whitney test, 0.01<p<0.05) relative to the basal level in C57BL/6 mice (grey bars), and ** indicates that these values are significantly different (Mann-Whitney test, 0.001<p<0.01) relative to the t-MeHA level measured at day 6 in H3R^−/−^ mice (black bars). *** indicates that HDC mRNA from H3R^−/−^ mice are significantly down-regulated as compared to that of C57BL/6 mice at day 6 (Mann-Whitney test, * p = 0.0336). (C) Determination of plasmatic histamine levels; histamineamia was measured by ELISA in naïve and infected C57BL/6 and H3R^−/−^ mice at different time points after inoculation with 10^6^ blood stage parasites of *Pb* ANKA. Values represent mean±standard deviation from three experiments. *A significant difference in histamineamia post infection between H3R^−/−^ mice and C57BL/6 mice were observed at day 3, and day 5 (Mann-Whitney test, p = 0.0117) only.

Once released from histaminergic neurons histamine is metabolized by two major enzymes, histamine-*N*-methyl transferase (HMT) and monoamine oxidase B [5], [17]. The HMT activity gives rise to *tele*-methylhistamine (t-MeHA), an inactive metabolite of histamine, the level of which accurately reflects neuronal histamine activity [5], [17]. To assess whether t-MeHA contents in the brains are differentially modulated by the parasites in C57BL/6 and H3R^−/−^ mice, these mice were inoculated with 10^6^ infected erythrocytes and their brains harvested at various time points after infection. As expected, given the regulatory role of H3 autoreceptors on histamine production, levels of t-MeHA in uninfected mice were significantly higher in H3R^−/−^ mice. At day 4 post-infection, there was a more dramatic decrease of the amounts of t-MeHA in H3R^−/−^ mice as compared to C57BL/6 mice (Fig. 5A). In H3R^−/−^ mice, the decrease in t-MeHA contents at all time points was significant as compared to the basal level. Also, at all time points tested, the levels of t-MeHA were higher in H3R^−/−^ than in C57BL/6 mice. These findings suggest the existence of either an infection-induced metabolism of histamine after its release from its cerebral stores or an infection-induced decrease of cerebral histamine synthesis.

To assess whether these changes in t-MeHA levels during infection reflect a regulation at the level of the biosynthetic pathway of histamine, we examined the level of expression of histidine decarboxylase (HDC), the enzyme that converts histidine into histamine. Kinetic analysis of HDC expression in the brain of C57BL/6 and H3R^−/−^ mice was performed at indicated times after the initiation of the infection until the expression of clinical signs of CM in H3R^−/−^ mice. As shown in figure 5B, the basal level of HDC mRNA was 3-fold higher in H3R^−/−^ mice than in C57BL/6 mice, a finding consistent with the higher level of t-MeHA in the brains of naive H3R^−/−^ mice (Fig. 5A). Until day 5 post-infection, this level remained relatively stable in H3R^−/−^ mice but at day 6, there was a marked decrease in the level of HDC expression. The regulation of the HDC expression was different in C57BL/6 mice where the HDC gene was significantly upregulated and was maintained at a constant level through day 6. It is striking to observe that, except for naive mice where the amounts of brain t-MeHA were well correlated with levels of HDC gene expression, in infected mice this correlation was poor in both mouse strains. This indicates that additional regulatory mechanisms associated with histamine metabolism are brought into play.

In an attempt to evaluate the impact of *Plasmodium* infection on serum histamine levels, we compared histamineamia of H3R^−/−^ mice and C57BL/6 mice infected or not with *Pb* ANKA. As shown in figure 5C, higher levels of histaminaemia were observed in infected H3R^−/−^ mice as compared to C57BL/6 mice only at day 3 and 5 (p = 0.0117) post-infection. This suggests an association between higher plasmatic levels of histamine and the severity of the disease in H3R^−/−^ mice, a parallel previously observed in murine models of CM [28] and in human infection [33]. These data also show that histamine levels are differentially regulated in peripheral compartments as compared to the brain.

### Splenic cytokine production during *Pb* ANKA infection

**Figure 6:**
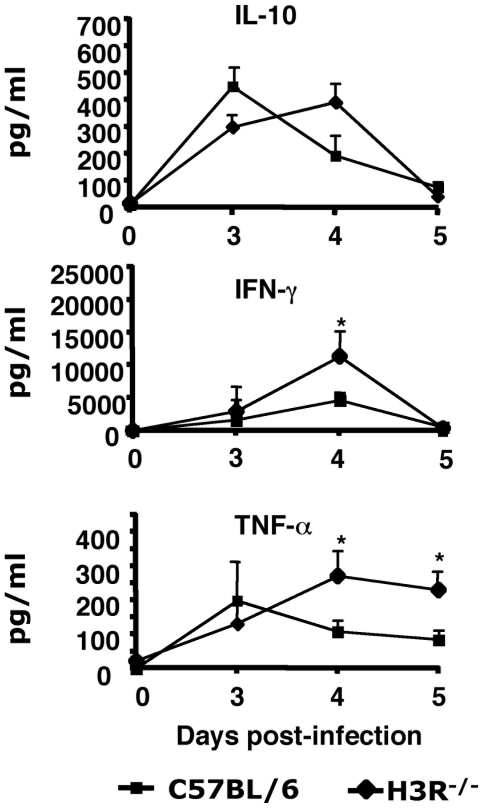
Antigen-specific T cell responses and cytokine analysis. Spleen cell preparations from infected H3R^−/−^ and C57BL/6 mice were seeded at 2×10^6^ cells/ml and incubated for 72 h in the presence or absence of 30 µg/ml of *Pb* ANKA lysate. After incubation, the supernantants were harvested and tested for their content in IL-10, TNF-α, and IFN-γ and measured by ELISA. * indicates that differences are significant (Mann-Whitney test, p = 0.039). Each experiment is representative of three distinct infections.

To explore the immune response of peripheral tissues during Pb *ANKA* infection, IL-10, IFN-γ, and TNF-α produced *in vitro* by *Pb* ANKA stimulated splenocytes from infected H3R^−/−^ and C57BL/6 mice were measured at various time points after infection. As shown in Fig. 6, all three cytokines could be elicited; IL-10 and TNF-α production peaked with a delay of one day in H3R^−/−^ mice as compared to C57BL/6 mice. Kinetically, the pattern of IFN-γ looks similar in the two mouse strains with however a 3-fold higher production (p<0.01) in H3R^−/−^ mice. At day 5, splenocytes from both mouse strains showed an inability to produce IL-10 and IFN-γ and a drastic reduction of TNF-α production upon antigenic challenge which at this time was maintained in H3R^−/−^ mice (Fig. 6). Since cytokines were induced in an antigen-dependent manner and no cytokines could be detected in antigen-stimulated unprimed splenocytes, cytokines were presumably produced by antigen-specific T cells. This pattern of time-dependent cytokine production, parallel to previous findings [34], was consistent with cell proliferation as measured with thymidine uptake (data not shown). These data indicate that the divergent susceptibility to CM of H3R^−/−^ and C57BL/6 mice could be a result of likely differences in T cell cytokine responses and thus supports a role for locally and peripherally dysregulated inflammatory response.

### A protective role for H3R agonists and H1R, and H2R inhibitors in CM

**Figure 7:**
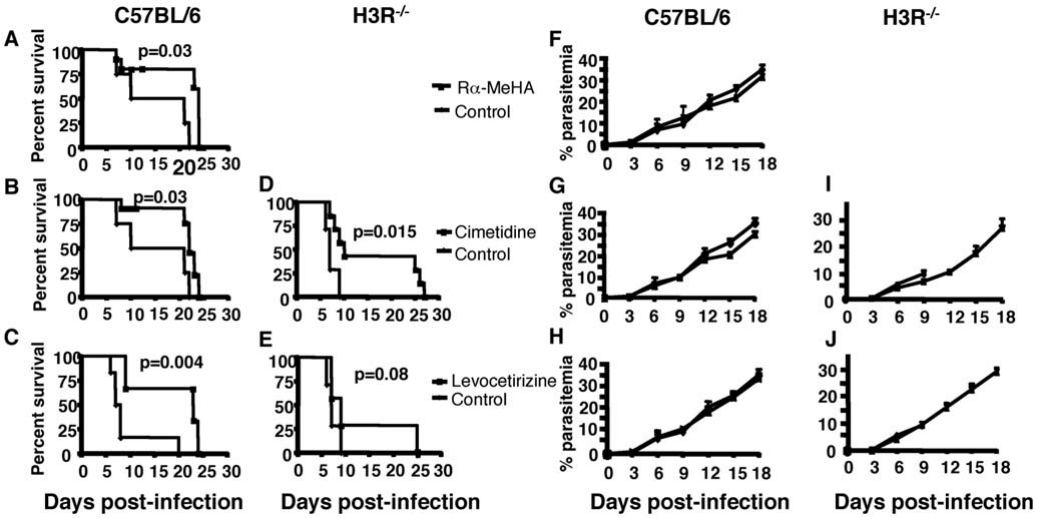
Effect of preventive treatment with histamine receptor agonists and antagonists on the occurrence of cerebral malaria. Wild-type C57BL/6 and H3R^−/−^ mice were left untreated (diamonds) or treated (squares) with a H3R agonist R-α-MeHA or either the H1R or H2R inhibitors, respectively, Levocetirizine, and Cimetidine, before and during infection with 10^6^ infected erythrocytes per mouse. These data are from two independent experiments. Significant differences in mortality/survival (A, B, C, D, E) were observed between drug-treated and untreated mice using the log-rank test. No significant differences in parasitemia between treated and untreated groups (F, G, H, I, J) were observed.

The higher susceptibility of H3R^−/−^ mice to CM suggests that histamine regulation via H3R has a protective effect in CM. To further assess the function of H3R during malaria disease, C57BL/6 mice were treated with (R)-alpha-methylhistamine, a standard H3R agonist [5], [17], and infected with *Pb* ANKA parasites. As shown in Figure 7A, administration of (R)-alpha-Methylhistamine had a significant protective effect with a median survival time of 24 days as compared with a median survival time of 15.5 days for untreated mice (n = 8, p = 0.029). Since we previously found that histamine signaling via H1R and H2R has a profound impact in malaria disease [28], and to verify that H1R and H2R inhibitors have additional beneficial effects to the naturally protective function of H3R, we examined the effect of Levocetirizine (H1R inhibitor) and cimetidine (H2R inhibitor) on CM in C57BL/6 mice. Administration of Cimetidine prolonged mouse survival with a median survival time of 22 days for treated mice as compared to a survival time of 15.5 days for untreated ones (n = 8, p = 0.03) (Figure 7B). Preventive treatment with Levocetirizine had a more protective effect against CM since median survival time was more than 25 days for treated mice as compared to a survival time of 8 days for untreated ones (Figure 7C). These data on median survival time demonstrate the shift from death by CM to mortality associated with hyperparasitemia mediated by pharmacologically decreasing histamine activity.

To further examine the efficacy of the preventive therapeutic effect of H1R and H2R inhibitors in the absence of the naturally protective H3R, H3R^−/−^ mice were treated with Levocetirizine and Cimetidine and inoculated with *Pb* ANKA parasite-infected erythrocytes. As shown in figure 7D, Cimetidine significantly prolonged the survival time (p = 0.015) whereas Levocetirizine was less effective (p = 0.08) (Figure 7E). Altogether, these data suggest that mimicking (H3R) or antagonizing (H1R and H2R) the biological effects of histamine may improve disease outcome in malaria infection. The fact that no difference in parasitemia between treated and untreated groups was observed suggests that the pharmacologic effects of histamine receptor inhibitors are exerted on host response rather than directly on the parasite.

**Figure 8:**
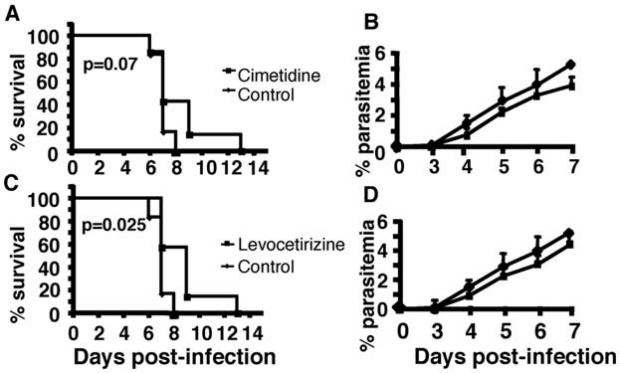
Effect of treatment with histamine receptor inhibitors on ongoing *Pb* ANKA infection. Panels A and C indicate survival rates of C57BL/6 infected with *Pb* ANKA 5 days prior daily treatments until the end of the experiment with either Cimetidine or Levocetirizine. These data are from one experiment using 7 mice per group. Significant differences in mortality/survival were observed between drug-treated and untreated mice using the log-rank test. Parasitemia (B, and D) were not significant.

To look whether histamine receptor inhibitors are able to protect mice once the infection is established, C57BL/6 mice were infected with *Pb* ANKA parasite-infected erythrocytes and beginning 5 days post infection mice were treated every day with either Levocetirizine or Cimetidine. As shown in figure 8A and C, Levocetirizine but not Cimetidine significantly increased mouse survival (p = 0.025, and p>0.05, respectively). Again, treatment with either drug appears to not effect parasite development directly since parasitemia in all treated groups was similar to that of control untreated mice (Figure 8B and D). Additional experiments where mice were treated using histamine H1 and H2 receptor inhibitors at the time of neurological sign manifestations did not show any therapeutic effect (supporting figure 2).

**Figure 9:**
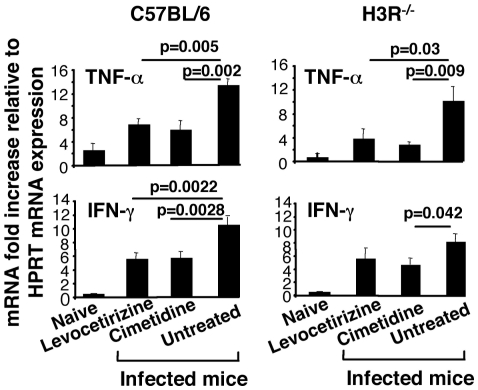
Down-regulation of inflammatory response-associated genes by histamine receptor antagonists. Wild-type C57BL/6 and H3R^−/−^ mice were treated or not with H1R inhibitor (Levocetirizine) or H2R inhibitor (Cimetidine) one day before and throughout the infection period by inoculating 10^6^ *Pb* ANKA-infected erythrocytes. Six days post-infection, brains were analyzed for the expression of TNF-α and IFN-γ as measured by quantitative RT-PCR. Gene mRNA expression is normalized relative to hypoxanthinephophoribosyltransferase. Data are presented as the means±SD from two independent experiments. Significant differences between groups indicated by presence of a bar over the respective columns were determined by Kruskal-Wallis test followed by Dunn's Multiple Comparison test.

We subsequently examined whether histamine effector functions via H1R and H2R alters expression of inflammatory genes in the brain in the context of functional (C57BL/6) or non functional H3R (H3R^−/−^). Analysis of mRNA expression of TNF-α and IFN-γ, two cytokines typically associated with CM, was performed by RT-PCR in the brain from C57BL/6 and H3R^−/−^ mice treated or not with Levocetirizine, or Cimetidine, and inoculated or not with 10^6^ *Pb* ANKA-infected erythrocytes. As shown in figure 9, a significant decrease of TNF-α (p = 0.0005, and 0.0002) and IFN-γ (p = 0.0022, and 0.0028) mRNA expression was observed in C57BL/6 mice treated with Levocetirizine, and with Cimetidine, respectively. In H3R^−/−^, mRNA expression of TNF-α was significantly reduced (p = 0.03, and p = 0.009) following treatment with Levocetirizine and Cimetidine, respectively whereas a significant down-regulation of IFN-γ gene expression was obtained only in mice treated with Cimetidine (p = 0.042). These data demonstrate that the therapeutic effect of H1R and H2R inhibitors appears to be more effective in the situation where H3R is functional (wild-type C57BL/6 mice) and can be interpreted at least in part by dampening the expression of inflammatory response-associated genes known to be critical for disease expression during CM.

## Discussion

It is widely recognized that the production of proinflammatory mediators and cytokines as well as the upregulation of endothelial cell adhesion molecules play an important role in the development and progression of cerebral pathology in malaria disease [35]. It has been hypothesized that CM is precipitated by the disruption of the brain microvasculature that results from the uncontrolled inflammatory reaction associated with inappropriate immune response [35]. During malaria pathogenesis, it is difficult to establish which comes first, the breakdown of BBB integrity or the pathogenic inflammatory response. We propose that resistance to CM development relies in the ability to control the inflammatory response that otherwise mediates immunopathological processes.

Our recent research was been based on the hypothesis that histamine is one of the earliest and potent inflammatory mediators that affects the host immune response to *Plasmodium* parasites. Recently, we tested this hypothesis by investigating the role of histamine in the occurrence of CM [28]. We found that HDC^−/−^ mice that lack histamine are resistant to the induction of CM during infection with *Pb* ANKA. In this mouse model, we found that the absence of histamine prevents leukocyte adhesion and sequestration of infected erythrocytes to the brain vasculature, and decreases the expression of ICAM-1 by brain vascular endothelial cells [28]. Given the predominant expression in the central nervous system of the H3R, one of the four identified histamine receptors, the possibility exists that signaling through this particular histamine receptor may control the development of *Plasmodium*-associated brain pathology. We investigated the role of H3R in the onset of CM, using mice with non-functional H3R gene. We observed a significantly earlier emergence of clinical signs of CM with more rapid death in H3R^−/−^ mice as compared to identically infected wild type C57BL/6 mice. Although higher parasitemia was observed at day 4 and 5 in H3R^−/−^ mice as compared to wild-type mice, it is unlikely that a causative relationship exists between parasitemia levels and pathology. Indeed, high parasitemia levels could be elicited in histamine-deficient HDC^−/−^ mice [28] and RAG^−/−^ mice (personal observation) without any disease expression. The accelerated death of H3R^−/−^ mice is associated with an earlier and more pronounced loss of BBB integrity than in infected C57BL/6 mice (Fig 2). These findings are consistent with an uncontrolled histamine release by histaminergic neurons in H3R^−/−^ mice. Indeed, histamine release by these neurons is tightly regulated by H3R, which act as inhibitory autoreceptors for the synthesis and release of histamine [5], [17] and as H3 heteroreceptors to inhibit the release of other neurotransmitters including dopamine, γ-aminobutyric acid, noradrenaline, acetylcholine, and serotonin in the CNS [18]. Estimating neuronal histamine levels in the brain, by measuring the histamine metabolite t-MeHA as an index, confirmed that neuronal histamine activity is significantly higher in naive H3R^−/−^ mice than in C57BL/6 mice. However, following infection, *tele*-methylhistamine levels in the brain dropped sharply below constitutive levels in H3R^−/−^ mice while in C57BL/6 mice this decrease was not obvious. This observation is consistent with a tighter control exerted by the H3R on histamine release from neuronal cells in C57BL/6 mice, which is absent in H3R^−/−^ mice. This decrease could be due either to a down-regulation of the HDC gene expression or an up-regulation of the HMT expression in response to the infection. We found that the catabolism pathway was not affected since the expression of HMT, which converts histamine into the biologically inactive compound *tele*-methylhistamine, was not altered throughout the infection period (data not shown). In naïve mice the biosynthetic pathway as determined by HDC mRNA expression indicates that expression in the brain was 3-fold higher in H3R^−/−^ mice than in C57BL/6 mice, a finding consistent with the control of histamine synthesis by H3 autoreceptors [5], [17]. After infection with *Pb* ANKA, the regulation of HDC mRNA expression showed distinct patterns in H3R^−/−^ and in C57BL/6 mice. It appears that while the expression of HDC increased in both mouse genotypes, at day 6 post-infection a down-regulation occurred only in H3R^−/−^ mice. A possible explanation for the earlier loss of BBB integrity in H3R^−/−^ mice consists of a surge in histamine available in the brain. This histamine likely binds to H1R and H2R which may then alter the permeability of brain capillaries resulting in the loss of BBB integrity. Our data show that the expression of clinical signs is associated with decreases in *tele*-methylhistamine levels, and HDC mRNA expression in the brain. However, it remains unclear by which mechanism these decreases occur in an accelerated manner in infected H3R^−/−^ mice. Because histamine neuron activity is involved in the regulation of body temperature [5] the earlier down-regulations observed in H3R^−/−^ mice might result as an effect of hypothermia [36], [37] known to occur during CM and just prior death. As part of the established neuroprotective effect of hypothermia, it was also reported very recently that hypothermia induced increased concentrations in the brain of galanin, a neuropeptide known to be co-localized with histamine in rodent histaminergic neurons and to modulate their activity [38]. Hence, an up-regulation of the neuropeptide galanin could provide a possible explanation [39], [40], [41]. Nonetheless, we could not detect differences in either the expression of galanin or in the drop of body temperature between H3R^−/−^ and C57BL/6 mice (data not shown). Given the complexity of the physiological regulations of a vast array of neurotransmitters by histamine via H3R signaling [38], future studies will explore if and how these neurotransmitters are modulated during *Plasmodium* infection in wild-type mice and in H3R^−/−^ mice.

In CM pathogenesis, it has been well established that a major effector is CD4^+^ and CD8^+^ T cells which sequester within cerebral blood vessels [42] along with parasitized red blood cells. Previous studies indicated that association of activated CD4^+^ and CD8^+^ T cells [42], [43], [44] and other inflammatory cells with the endothelial cells of brain vasculature is a characteristic of experimental CM. We examined whether the higher susceptibility of H3R^−/−^ mice was reflected by a differential recruitment of these cells as compared to wild type C57BL/C mice. Analysis of brain-associated CD4^+^ and CD8^+^ T cells after infection with *Pb* ANKA parasites displayed a constant rise in T cells with similar kinetics in both genotypes until day 5 where a higher number of cells was detected in the brains from H3R^−/−^ mice. Considering the fact that other inflammatory cells such as neutrophils [45], [46] and monocytes [47] contribute to CM pathogenesis, we evaluated and observed, as for T cells, a sequestration of a higher number of cells in the brains of infected H3R^−/−^ mice. Furthermore, T cell sequestration was dominated by CD8^+^ T cells. Looking at a possible difference in terms of peripheral T cell response, the splenocytes from the mouse strains, challenged ex-vivo with *Pb* ANKA lysate, produced variable amounts of IL-10, IFN-γ and TNF-α with different kinetics between the two genotypes ultimately ending with an immunosuppression at day 5 post-infection occurring in both mouse strains. This immune suppression is consistent with earlier data [34], further suggesting that inhibition of the T cell proliferation and cytokine production was under the control of CD4^+^CD25^+^ T regulatory cells elicited during *Pb* ANKA infection. One exception for this immune suppression is TNF-α whose production by splenocytes from H3R^−/−^ mice was less affected. The sustained TNF−α production by splenocytes combined with a sequestration of a higher number of CD4^+^ and CD8^+^ T cells and increased production of pro-inflammatory cytokines in the brain may account for the more severe disease developed by H3R^−/−^ mice.

A critical and consistent difference between the two mouse genotypes is the earlier disruption of the BBB and the concomitant hemorrhagic lesions in H3R^−/−^ mice at day 3 post-infection, a time when no clinical sign had manifested.

Histamine is implicated in the pathophysiology of multiple sclerosis and its animal models, collectively termed EAE [48]. Our findings are in agreement with a recently published report on EAE showing that, compared with wild-type animals, H3R^−/−^ mice develop a more severe disease and neuroinflammation along with dysregulated BBB permeability [48]. Typically, the increased clinical disease observed during the early phase of EAE in H3R^−/−^ mice was associated with earlier and more severe inflammatory infiltrates. Also, the degree of the BBB permeability was significantly higher in H3R^−/−^ compared with C57BL/6 mice. As for EAE, two opposite, although not exclusive hypotheses, may account for the pathogenetic mechanisms involved in the disease expression during experimental CM. The immune-based hypothesis which contends that parasite-specific CD4^+^ and CD8^+^ T cells exert their effector functions within brain capillaries, and the alternative hypothesis that emphasizes the brain tissue-initiated inflammatory response, which represents the primary pathological event leading to disease progression. Disruption of H3R reveals that during *Pb* ANKA infection, the ability of this receptor to normalize histamine release within the brain tissue is important for limiting immunopathology and supporting a positive outcome. Subsequent to the brain-initiated inflammatory events (Fig. 2, and 3), CD4^+^ and CD8^+^ T cells and inflammatory cells adhere to brain capillaries leading to their detrimental effect. In this regard, we propose a similar chronological pattern of pathogenetic events as in EAE and as discussed by Teuscher et al. [48].

Uncontrolled histamine release in various immunopathological conditions where the brain tissue represents the site of pathogenetic events may support the utility of pharmacological targeting of H3R via receptor agonists to prevent the development of tissue lesions either in multiple sclerosis or during CM. Our data support this pharmacological approach since the use of (R)-alpha-methylhistamine, a standard H3R agonist [5], [17], was demonstrated to be effective in reducing progression to CM. Although, such class of drug is not available yet for a therapeutic use in humans, our work provides a rational basis for a future use of H3R agonists, alone or in combination with H1R or H2R inhibitors, as they become available. The single use, however, of an H1R inhibitor Levocetirizine or an H2R inhibitor Cimetidine when H3R is functional appears to be promising since both drugs were effective in preventing the development of clinical signs and mortality due to CM. This therapeutic effect was translated at the molecular level at least in part by the down-regulation of inflammatory response-associated genes such as IFN-γ and TNF-α. Hence, the beneficial role of H3R in limiting disease expression by controlling histamine release and metabolism is highlighted by the higher therapeutic effectiveness and better control of inflammatory gene expression exerted by H1R and H2R inhibitors when H3R is functional. The compensatory protective effect provided by Cimetidine and to a lesser extent by Levocetirizine in H3R^−/−^ mice suggest that expression of a functional H3R constitutes a minimal condition that helps slow the development of severe disease. Once the infection was established, we found that only Levocetirizine significantly increased survival to CM. Furtheremore, Levocetirizine appeared to be more efficient when delivered as a preventive treatment than when it was administered therapeutically. This argues for a preferential use of H1R and H2R inhibitors as a preventive therapy. This would be more feasible in areas where malaria transmission is seasonal. In the absence of any impact or alteration of parasite development, our data are not consistent with previous findings showing that Astemizole, an H1R inhibitor, directly interferes with *Plasmodium* parasite metabolism, thus altering its development [49]. Our previous and current data with histamine support a mechanism by which anti-histamines administered during infection with *Pb* ANKA affect the host immune response by maintaining the inflammatory response at levels that are less detrimental to the host and thereby reducing progression to CM.

## Materials and Methods

### Ethics statement

All animal care and experimentation were conducted in accord with Pasteur Institute animal care and use committee guidelines.

### Mice

Female C57BL/6 mice 6–8 wk old were purchased from Charles River Breeding Laboratories (Saint-Aubin les Elbeufs, France). H3R^−/−^ mice [50] were provided by Dr Robin L. Thurmond (Johnson & Johnson Pharmaceutical Research and Development, San Diego, California). All knockout mice originated from the C57BL/6 background.

### Parasites and infection

For all infections, a cloned line of *Plasmodium berghei* ANKA (*Pb* ANKA) strain stably transfected with the green fluorescent protein (GFP) on hsp70 promoter [51], was utilized allowing the detection of sporozoites and blood stage parasites by fluorescent microscopy. *Pb* ANKA was provided by Dr. T. Ishino (Department of Medical Zoology, Mie University School of Medicine, Edobashi, Tsu, Japan). Infection of mice induces experimental CM, characterized by paralysis, ataxia, convulsions and coma between 6–11 days post-infection. This parasite was maintained in a cycle between C57BL/6 mice and *An. stephensi* [52]. The erythrocytic stages of the parasite were maintained in liquid nitrogen as parasitized red blood cells in Alsever's solution (Sigma, France) containing 10% glycerol. The infection was induced by intraperitoneal injection of 10^6^ parasitized red blood cells.

### Preparation of brain cell suspensions

The brains were obtained from H3R^−/−^ mice at the coma stage of CM and at the same time from wild-type C57BL/6 mice (day 6). Briefly, mice were anesthesized with ketamine (600 mg/kg) and xylazine (20 mg/kg) and perfused with 30 ml of PBS via the left ventricle of the heart to remove circulating and nonadherent RBC and leukocytes from the brain vasculature. Brain-associated leukocytes were obtained as described previously [28].

### Flow cytometric analysis of brain leukocytes

Brain cells were stained for FACS analysis according to standard protocols in cold PBS containing 2% FCS and 0.01% sodium azide (FACS buffer) with the following Abs (all from BD Biosciences, Le Pont de Claix, France): FITC-labeled anti CD45, APC-labeled CD4, phycoerythrin (PE)-labeled anti-CD8α, PE-labeled anti-GR1, and APC-labeled anti-CD11b. After staining with Abs, cells were washed then resuspended in FACS buffer before flow cytometric analysis. A total of 5×10^4^ living cells for brain, gated as CD45-positive cells, were analyzed using a four-color FACSCalibur flow cytometer with ProCellQuest software (BD Biosciences, Mountain View, California).

### Permeability of the blood-brain barrier

When mice infected with *Pb* ANKA strain began showing neurological symptoms, usually at day 6–7 post-infection. A volume of 200 µL of 2% (w/v) solution of Evans Blue in PBS was injected into the mice retro-orbitally. One hour later, mice were perfused with PBS after anesthesia with ketamine (600 mg/kg) and xylazine (20 mg/kg). Each brain was removed and photographed.

### Animal perfusion and histological analysis

To analyze only brain-associated leukocytes, mouse perfusion consisted of intracardiac injection of 100 ml PBS, followed by 200 ml of 4% paraformaldehyde in PBS. The brains were fixed for 3 days in 1% paraformaldehyde solutions and then were subsequently cryoprotected in a 30% sucrose phosphate-buffered solution at 4°C for 3 days before cutting. For May-Grünwald Giemsa staining procedure, the protocols used to obtain brain sections (40 µm thick) were described elsewhere [53], [28]. Although sequestration of infected erythrocytes occurs evenly without any particular localization in the brain tissue (infected erythrocytes were found in olfactory bulbs, cortex, thalamus, hypothalamus, and cerebellum), sections were made in three different mice from the same anatomical sites of the cerebellum only. Detection of blood-stage parasites associated with sequestered erythrocytes in the brain could be visualized either by May-Grünwald Giemsa staining or GFP fluorescence. The density of erythrocyte aggregates in brain sections of infected and control mice were expressed as the average of aggregates per field and counted in 100 consecutive microscopic fields from 3 histological sections per brain at magnification 250×. Images of randomly selected were collected using an Olympus upright microscope (BX61) (Center Valley, Pennsylvania) equipped with an oil-immersion lens (60×) and a cooled video camera (Qimaging, Retiga 2000R, Burnaby, Canada) with a color conversion filter. Digitizing was performed with a PC computer using the image analysis system Image-Pro Plus (MediaCybernetics, Silver Spring, USA).

### Splenocyte proliferation assay and cytokine production

Spleen cells from C57BL/6 or H3R^−/−^ mice were collected at different time points after infection with *Pb* ANKA and incubated for 72 h in the presence or absence of 30 µg/ml of *Pb* ANKA lysate. For proliferation assays, 200 µl total splenocyte suspensions, diluted in complete RPMI 1640 medium–5% fetal calf serum, were seeded in 96-well plates at a density of 2×10^6^ cells/ml. Cells cultured in medium alone were used as background controls. [methyl-^3^H]thymidine (2 µCi/well; Amersham Biosciences, United Kingdom) was added 16 h before harvesting, and radioactivity was measured using a Betaplate counter. For cytokine detection, the cell culture supernatants were collected and analyzed for their cytokine content by capture enzyme-linked immunosorbent assay (ELISA). Detection of IL-10, IFN-γ, and TNF-α was performed according to the manufacturer's instructions (BD Biosciences, Mountain View, California).

### Quantification of brain-associated HDC, HMT, and immune signaling molecules

Gene expression in the brains from H3R^−/−^ and C57BL/6 mice at various time points post-infection was analyzed by the real-time RT-PCR. RNA utilized for these assays was isolated by means of a two-step extraction process. First, brains were surgically removed from mice as previously described and placed immediately in RNAlater at 4°C overnight. After RNAlater infused the samples, it was removed and samples were maintained at −80°C until processing. Brains were thawed in 1 ml of Trizol and subjected to bead disruption in a polytron 3 times from 2 min at a setting of 30 cycles/sec. Samples were spun at high speed (10,000×g) for 3 min to remove debris and lipids. Half of the sample was transferred to a new tube and mixed with 500 µl of Trizol reagent by vortexing. Following this step, RNA extraction proceeded according to manufacturers protocol. Precipitated RNA was resuspended in 100 µl of RNase-free water. The second step of this extraction was followed by Qiagen's protocol for RNA clean-up including steps for removal of protein and DNA (Qiagen RNeasy Kit). Samples were eluted with 50 µl of RNase-free water and quality and quantity assured by photospectroscopy. Real-time RT-PCR utilized various primer-probe sets and standard Taqman protocols (Applied Biosystems) [54].

### Determination of tele-Methylhistamine Levels in brain

After infection, animals were sacrificed and the brain was homogenized in 10 volumes (w/v) of ice-cold perchloric acid (0.4 N). t-MeHA levels in the supernatants were determined using an enzymoimmunoassay derived from a radioimmunoassay described previously [55]. Briefly, t-MeHA of the sample was derived with p-benzoquinone (BZQ) (2.8 mg/ml). The reaction was allowed to proceed at pH 7.9 for 3 h, then 2 M glycine was added to eliminate the excess of p-benzoquinone. The extract was mixed with t-MeHA-p-benzoquinone-Leu-Tyr-acetylcholinesterase as a tracer and an antiserum raised in rabbits against t-MeHA conjugated with bovine serum albumin via p-benzoquinone in a 96-well plate (Nunc Immuno-Plate Maxi-Sorp Surface; Nunc, Roskilde, Denmark) pretreated with swine anti-rabbit IgG (Cayman Chemical, Ann Arbor, MI). After incubation for 16 h at 15°C, plates were washed and the substrate for acetylcholinesterase, Ellmann's reagent, was added. After 5 h, the optical density was measured with a Dynatech Mr 5000 at 405 nm. The limit of the detection was 5 pg of t-MeHA.

### Treatments

The histamine receptor subclass-specific inhibitors Cimetidine (H2R), and Levocetirizine (H1R) and the H3R agonist (R)-alpha-methylhistamine (R-α-MeHA) were injected i.p. daily at 10 mg/kg until the end of the experiment, starting 24 h before infection. To test the therapeutic effect on histamine receptor inhibitors, in one experiment, Cimetidine and Levocetirizine were injected daily from day 5 post-infection until the end of the experiment. Levocetirizine was kindly obtained from UCB Biopharma (Brussels, Belgium), whereas Cimetidine and R-α-MeHA were purchased from Sigma (St Louis MO). Histamineamia was determined using ELISA kits purchased from Neogen Corporation (Lexington, KY).

### Statistical analysis

Significant differences in survival were evaluated by generation of Kaplan-Meier plots and log rank analysis. *p*<0.05 was considered statistically significant. For other analyses, when differences between C57Bl/6 and H3R-KO mice were compared at a given time point, the Mann-Whitney test was performed with significance set at p<0.05. When comparing differences between days after inoculation within each strain of mice, we performed the Kruskal-Wallis test followed by an *a posteriori* Dunn's multiple comparison test.

## Supporting Information

Figure S1 Morphological characterization of the CD11b+GR1 high cell population. This cell population could represent a population of GR1+ inflammatory monocytes that are elicited during Plasmodium parasite infection. We have reassessed cellular analysis using anti-GR1 Ab combined with the 7/4 mAb (Caltag Laboratories) which reacts with the 7/4 antigen that is a polymorphic 40 kD molecule expressed by polymorphonuclear cells, but absent on resident tissue macrophages. This analysis is shown in S1A. A similar pattern was obtained as previous experiments based on high expression of GR-1 epitope. However, the 7/4 antigen can also be expressed by inflammatory monocytes as shown in S1D with a key difference in that inflammatory monocytes express less GR1 and 7/4 antigen than neutrophils. We further examined these cells which were sorted using a cell-sorter (5 days post-infection) as shown in annex 1B (purity 96% based on GR1High/7/4 expression) and which morphologically resemble activated neutrophils as observed by electron microscopy (S1C). These cells were also shown to release myeloperoxydase upon phorbol myristate acetate stimulation.
(1.10 MB TIF)

Figure S2 Treatments with histamine receptors inhibitors have no therapeutic effect when administered at the time of CM neurological symptoms. C57BL/6 mice were first infected with 106 parasitized erythrocytes and when the mice showed signs of cerebral malaria at day 7 (60 to 80% of the mice), they received daily either Levocetirizine or Cimetidine.
(0.25 MB TIF)
