# Supplementary figures and images for: Histamine H3 Receptor-Mediated Signaling Protects Mice from Cerebral Malaria
Source: PLoS One. 2009 Jun 23;4(6):e6004. doi: 10.1371/journal.pone.0006004 (PMC2696087; doi:10.1371/journal.pone.0006004)

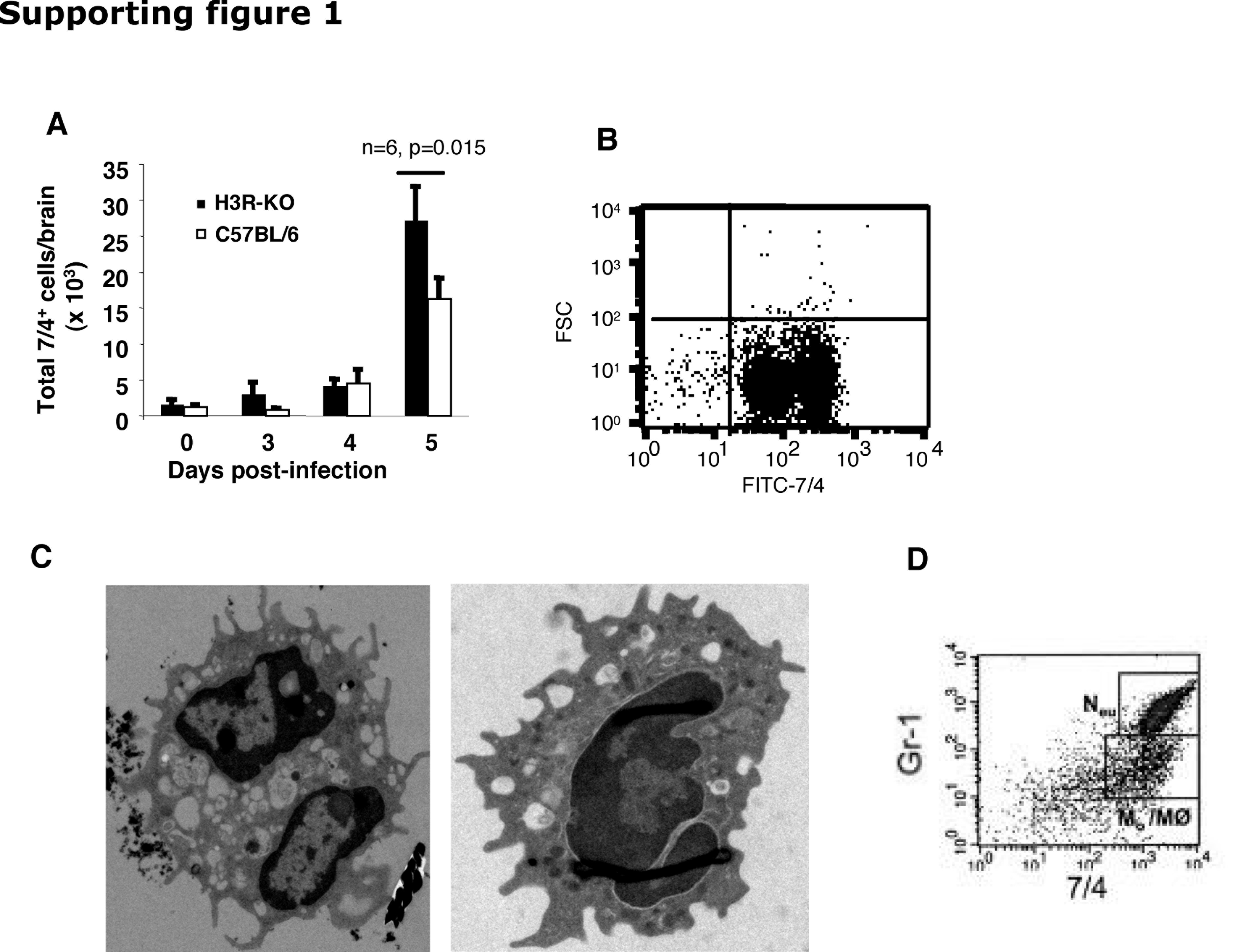

Supplement: Figure S1 — Morphological characterization of the CD11b+GR1 high cell population. This cell population could represent a population of GR1+ inflammatory monocytes that are elicited during Plasmodium parasite infection. We have reassessed cellular analysis using anti-GR1 Ab combined with the 7/4 mAb (Caltag Laboratories) which reacts with the 7/4 antigen that is a polymorphic 40 kD molecule expressed by polymorphonuclear cells, but absent on resident tissue macrophages. This analysis is shown in S1A. A similar pattern was obtained as previous experiments based on high expression of GR-1 epitope. However, the 7/4 antigen can also be expressed by inflammatory monocytes as shown in S1D with a key difference in that inflammatory monocytes express less GR1 and 7/4 antigen than neutrophils. We further examined these cells which were sorted using a cell-sorter (5 days post-infection) as shown in annex 1B (purity 96% based on GR1High/7/4 expression) and which morphologically resemble activated neutrophils as observed by electron microscopy (S1C). These cells were also shown to release myeloperoxydase upon phorbol myristate acetate stimulation. (1.10 MB TIF) [file pone.0006004.s001.tif]

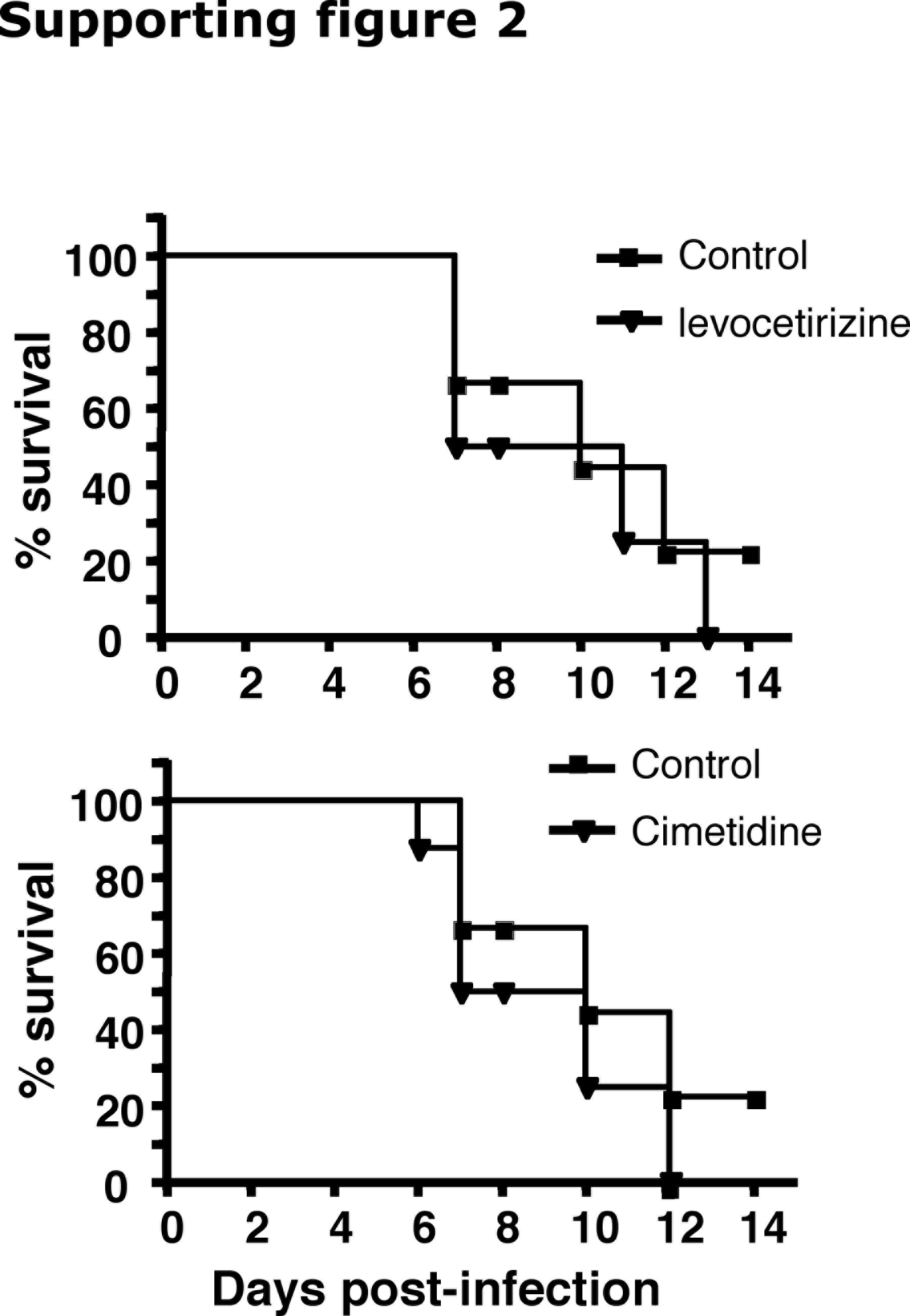

Supplement: Figure S2 — Treatments with histamine receptors inhibitors have no therapeutic effect when administered at the time of CM neurological symptoms. C57BL/6 mice were first infected with 106 parasitized erythrocytes and when the mice showed signs of cerebral malaria at day 7 (60 to 80% of the mice), they received daily either Levocetirizine or Cimetidine. (0.25 MB TIF) [file pone.0006004.s002.tif]
